# HER2-Displaying M13 Bacteriophages induce Therapeutic Immunity against Breast Cancer

**DOI:** 10.3390/cancers14164054

**Published:** 2022-08-22

**Authors:** Junbiao Wang, Alessia Lamolinara, Laura Conti, Mara Giangrossi, Lishan Cui, Maria Beatrice Morelli, Consuelo Amantini, Maurizio Falconi, Caterina Bartolacci, Cristina Andreani, Fiorenza Orlando, Mauro Provinciali, Francesco Domenico Del Pizzo, Francesca Russo, Barbara Belletti, Federica Riccardo, Elisabetta Bolli, Elena Quaglino, Federica Cavallo, Augusto Amici, Manuela Iezzi, Cristina Marchini

**Affiliations:** 1 School of Biosciences and Veterinary Medicine, Biology Division, University of Camerino, via Gentile III da Varano, 62032 Camerino, Italy; 2 Center for Advanced Studies and Technology, Department of Neurosciences, Imaging and Clinical Sciences, G. d’Annunzio University of Chieti-Pescara, 66013 Chieti, Italy; 3 Department of Molecular Biotechnology and Health Sciences, Molecular Biotechnology Center “Guido Tarone”, University of Torino, 10126 Torino, Italy; 4 School of Pharmacy, University of Camerino, 62032 Camerino, Italy; 5 Experimental Animal Models for Aging Unit, Scientific Technological Area, IRCCS INRCA, 60100 Ancona, Italy; 6 Molecular Oncology Unit, Centro di Riferimento Oncologico di Aviano (CRO Aviano), IRCCS, National Cancer Institute, 33081 Aviano, Italy; 7 Department of Life Sciences, University of Trieste, 34128 Trieste, Italy

**Keywords:** bacteriophages, breast cancer, cancer vaccines, HER2, Δ16HER2, anti-tumor immunity

## Abstract

**Simple Summary:**

The high incidence and death rates of breast cancer make the development of new therapies an urgent need. The introduction into the clinic of the anti-HER2 monoclonal antibody trastuzumab considerably improved the overall survival and time-to-disease progression of patients with HER2-positive breast cancer. However, many patients do not benefit from it because of resistance to therapy. Cancer vaccines, by inducing into the patient an anti-cancer specific immunity, might represent an alternative immunotherapeutic approach, but despite promises, so far no anti-HER2 cancer vaccine has been approved for human use. In this study, we propose therapeutic phage-based vaccines, against HER2 and its aggressive isoform Δ16HER2, able to elicit a protective immunity and potentially capable of preventing relapse in HER2-positive breast cancer patients, even in those who develop trastuzumab resistance.

**Abstract:**

The advent of trastuzumab has significantly improved the prognosis of HER2-positive (HER2+) breast cancer patients; nevertheless, drug resistance limits its clinical benefit. Anti-HER2 active immunotherapy represents an attractive alternative strategy, but effective immunization needs to overcome the patient’s immune tolerance against the self-HER2. Phage display technology, taking advantage of phage intrinsic immunogenicity, permits one to generate effective cancer vaccines able to break immune tolerance to self-antigens. In this study, we demonstrate that both preventive and therapeutic vaccination with M13 bacteriophages, displaying the extracellular (EC) and transmembrane (TM) domains of human HER2 or its Δ16HER2 splice variant on their surface (ECTM and Δ16ECTM phages), delayed mammary tumor onset and reduced tumor growth rate and multiplicity in ∆16HER2 transgenic mice, which are tolerant to human ∆16HER2. This antitumor protection correlated with anti-HER2 antibody production. The molecular mechanisms underlying the anticancer effect of vaccine-elicited anti-HER2 antibodies were analyzed in vitro against BT-474 human breast cancer cells, sensitive or resistant to trastuzumab. Immunoglobulins (IgG) purified from immune sera reduced cell viability mainly by impairing ERK phosphorylation and reactivating retinoblastoma protein function in both trastuzumab-sensitive and -resistant BT-474 cells. In conclusion, we demonstrated that phage-based HER2 vaccines impair mammary cancer onset and progression, opening new perspectives for HER2+ breast cancer treatment.

## 1. Introduction

Human epidermal growth factor receptor 2 (HER2) tyrosine kinase receptor is overexpressed in 20–30% of all breast cancer (BC) cases and is associated with poor prognosis. Trastuzumab/Herceptin^TM^, a humanized monoclonal antibody targeting the extracellular domain of HER2, is one of the most successful targeted therapies for HER2-overexpressing BC. HER2 dimerization enhances cell motility, survival, and proliferation mainly through activation of the MAPK/ERK and the phosphatidylinositol 3-kinase (PI3K)-Akt signaling pathways. When trastuzumab binds to HER2, it induces a reduction in cell proliferation by interfering with these signaling pathways, and by inducing a downregulation of HER2 through endocytosis [1,2]. Antibody-Dependent Cellular Cytotoxicity (ADCC) is also crucial for the in vivo anti-tumor function of trastuzumab [3,4]. However, although trastuzumab significantly improves survival outcomes for HER2+ BC patients, both de novo and acquired resistance represents a still unsolved problem [5]. Furthermore, trastuzumab has been associated with significant risk of cardiotoxicity, which increases significantly with age [6]. In this scenario, cancer vaccines are the next goal to reach in the battle against BC. They are conceived to confer a long-term protection from recurrences and metastases by inducing into the patient a self-production of anti-cancer specific antibodies and cell-mediated immunity. Despite encouraging preclinical results [7,8], BC vaccines have been unsuccessful when tested in clinical trial [9] and therapeutic vaccines for HER2+ BC patients are not available yet. The lack of significant clinical benefits is mainly related to immune-suppressing and tumor-evading mechanisms developed by advanced tumors and to the immunological tolerance to self-antigens, as it is the HER2. One of the most promising strategies to enhance the immunogenicity of anti-HER2 vaccines is provided by phage display technology [10]. Indeed, viral particles, which are intrinsically immunogenic, can be exploited to expose antigenic proteins fused to phage capsid proteins in order to overcome the suppressive and tolerogenic tumor environment and, at the same time, to induce a specific immune response. Moreover, bacteriophages can infect and replicate within bacteria but are incompetent for eukaryotic infection; thus, they have a unique potential as biotechnological tools with a good safety profile. The most common bacteriophages used in phage display are M13 and fd filamentous phages [11,12]. M13 are non-lytic viruses that specifically infect *Escherichia coli* (*E. coli*) cells showing an F pilus. They consist of a flexible protein-based cylinder assembled around a circular single-stranded DNA genome and are considered powerful systems for the development of vaccines. Indeed, they: (a) can be easily engineered to present antigens on their surface; (b) can evoke both innate and adaptive immune responses against displayed antigens, stimulating both cellular and humoral immunity; (c) are considered safe for administration in humans [13,14]. In particular, filamentous bacteriophages are capable of being taken up by antigen-presenting cells and processed efficiently by MHC class I and class II pathways, generating both Cytotoxic T-lymphocyte (CTL) and T helper responses [15]. Moreover, phage stability can facilitate phage-based vaccine transport and storage. Such properties make recombinant bacteriophages ideal vaccination platforms for cancer immunotherapy. Preclinical evidence indicates that phage-based vaccines are effective against melanoma [16,17], Lewis lung carcinoma [18], BC [19,20] and lymphoma [21]. A clinical trial for a filamentous phage-based vaccine exposing a selected idiotype (B-cell receptor-Ig variable region) against multiple myeloma demonstrated that this kind of vaccination is well-tolerated, immunogenic and able to induce a clinical response in most patients [22]. We recently developed anti-HER2 phage-based vaccines by engineering M13 filamentous phages in order to make them display the extracellular (EC) and transmembrane (TM) domains of human HER2 (ECTM) or its Δ16HER2 splice variant (Δ16ECTM) or selected HER2 immunogenic epitopes [23]. The expression of the Δ16HER2 isoform, lacking exon 16, has been frequently detected in HER2+ human BC and correlates with metastatic disease [24,25]. The high oncogenic potential of Δ16HER2 is due to its ability to form constitutively active homodimers, stabilized by disulphide bonds, and thus to trigger sustained proliferative signaling. Here, we investigated the preventive and therapeutic efficacy and immune reactions triggered by phage-based vaccines in Δ16HER2 female mice, which develop spontaneous aggressive mammary adenocarcinomas (luminal B subtype), starting from 11 to 12 weeks of age, due to the expression of the human Δ16HER2 transgene in mammary epithelial cells [26,27,28], and are immuno-tolerant toward the human HER2 [23], mimicking what is encountered clinically. We demonstrated that the addition of HER2 immunogenic sequences to the minor coat protein III (pIII) of M13 phage particles breaks tolerance against HER2 self-protein and elicits protective anti-cancer immune responses when administered before tumor onset in ∆16HER2 transgenic mice. Moreover, we reported that phage-based vaccination can control the growth of mammary carcinomas in Δ16HER2 mice even when employed in a therapeutic setting. Phage-vaccine anticancer efficacy correlates with the induction of anti-HER2 antibodies, which are able to interfere with HER2 downstream signaling pathways and to reduce cell viability in vitro even in trastuzumab-resistant BC cells. Thus, anti-HER2 phage-vaccination might represent a promising alternative for HER2+ breast cancer patients who cannot benefit of trastuzumab treatment due to the development of drug resistance.

## 2. Materials and Methods

### 2.1. Phages Production and Purification

To produce recombinant phages, pIF6-phagemid harboring HER2 or Δ16HER2 fragments (Δ16ECTM, and ECTM fused with gIIIp of M13 phage (NEB) [23]) were used to transform *Escherichia coli* TG1 cells. TG1 cells containing phagemids were grown overnight in 2x TY medium with 100 µg/mL ampicillin and 1% glucose at 37 °C. When optical density (OD) measurement at 600 nm reached 0.4–0.5, referring to the log phase of growth, phagemid-transformed TG1 cells were superinfected with the helper phage M13K07 at a multiplicity of infection (MOI) of 20:1, and incubated for 30 min at 37 °C without shaking and then 30 min at 37 °C with shaking. Bacterial cultures were then centrifuged for 10 min at 2465× *g*. The pellet of TG1 cells was resuspended in 2x TY medium, additioned with 100 µg/mL ampicillin, 70 µg/mL kanamycin and 200 µmol/L of Isopropyl β-d-1-thiogalactopyranoside (IPTG) and grown overnight at 30 °C. Phages were purified using PEG-based precipitation. M13 recombinant phages were harvested from the supernatant after centrifugation of the infected culture for 30 min at 352× *g*, then phages were precipitated with 3:10 *v*/*v* ratio of PEG (polyethylene glycol)—NaCl (20% PEG, 2.5 M NaCl) through incubation for 2 h in ice, pelleted by centrifugation at 4 °C for 2 h at 3220× *g* and suspended in 2 mL of PBS. They were subsequently filtered through a 0.22 µm Millipore filter and then titrated by top agar plaque assay in order to ultimately quantify recombinant virion particles. The concentration of virion particles was verified by UV absorption spectrometry using the formula:

virions/mL = [(A269 − A320) × 16 × 10^16^]/(number of bases/virion)

### 2.2. Mice

Δ16HER2 transgenic mice and FVB mice (FVB/NCrl strain from Charles River) were housed under controlled temperature (20 °C) and circadian cycle (12 h light/12 h dark). The animals were fed on a chow diet (Mucedola) and tap water ad libitum. Mice were treated in accordance with the U.K. Animals (Scientific Procedures) Act, 1986, and associated guidelines, EU Directive 2010/63/EU for animal experiments and with the 3Rs principles. All animal experiments were authorized by the Italian Ministry of Health (#516/2019-PR) and by the Animal Research Committee of the University of Camerino (OPBA).

### 2.3. Phage Immunization

Δ16HER2 transgenic females were divided into three experimental groups (9–11 mice/group), each receiving distinct phage preparations (ECTM phages, or Δ16ECTM phages or empty phages as control). 10^10^ PFU/mouse in 0.1 mL PBS were intraperitoneally (i.p.) injected at 8, 10 and 12 weeks of age (preventive vaccination) or at 12, 14 and 16 weeks of age (therapeutic vaccination). Blood was collected from the retro-orbital plexus under anesthesia before and 2 weeks after the last boost to verify the presence of HER2-specific antibodies. Vaccinated animals were weekly monitored for tumor onset, growth, and multiplicity by palpation until 24 weeks of age. Progressively growing masses ≥2 mm mean diameters were regarded as tumors. Two perpendicular diameters (a and b) were measured on each tumor using caliper and volumes were calculated by the V = π/6[(a + b)/2]^3^ formula.

### 2.4. Purification of IgG Elicited by Vaccination

IgG were purified from sera of immunized mice using the Melon Gel IgG Purification Kit (Thermo Fisher Scientific, Waltham, MA, USA) and quantified using NanoDrop spectrophotometer using IgG settings (Thermo Fisher Scientific, Waltham, MA, USA).

### 2.5. Cell Lines

Human BT-474 cells were obtained from American Type Culture Collection (Rockville, MD, USA) and cultured in Dulbecco’s Modified Essential Medium (DMEM, Gibco, Thermo Fisher Scientific, Waltham, MA, USA) supplemented with 10% fetal bovine serum (FBS, Gibco, Life Technologies) and 1% penicillin–streptomycin (P/S) (Gibco, Life Technologies). CAM3 and CAM6 cells, Δ16HER2-expressing epithelial tumor cell lines established from a mammary carcinoma spontaneously arisen in a Δ16HER2 female [29], were cultured in DMEM supplemented with 20% FBS and 1% P/S. Δ16HER2-HEK293 cells, a generous gift from the Unit Department of Experimental Oncology-Istituto Nazionale Tumori di Milano, were maintained in G418 antibiotic (Gold Biotechnology, Saint Louis, MO, USA; 1 mg/mL). Cells were maintained at 37 °C in an atmosphere of 5% CO_2_.

### 2.6. Establishment of BT-474 Trastuzumab-Resistant BC Cell Line (Acquired Resistance)

The trastuzumab-resistant BT-474.R cell line was established by culturing the BT-474 cell line in the appropriate medium supplemented with 15 μg/mL of trastuzumab (Herceptin, Genentech, San Francisco, CA, USA) for 8 months as described by Zazo S. et al. [30]. Cells were grown at 10 μg/mL trastuzumab in culture medium for 30 days, at which point the dose was increased to a final concentration of 15 μg/mL. Once the establishment of resistance was confirmed, the cells were kept at a 15 μg/mL maintenance dose.

### 2.7. Cell Viability Assay

The effects of control- or immune-IgG or trastuzumab on cell viability were assessed by seeding 8000 cells/well (trastuzumab-sensitive and -resistant BT-474 cells), and 2 × 10^4^ cells/well (CAM6 cells) in 96-well plates in DMEM plus 2% FBS. The day after, fresh medium containing appropriate IgG or trastuzumab concentrations was added. Cell viability was determined after 72 h using an MTT (3-(4,5-dimethylthiazol-2-yl)-2,5-diphenyl-2H-tetrazolium bromide, Sigma Aldrich/Merck, Darmstadt, Germany) assay, which is based on the conversion of MTT to formazan by mitochondrial enzymes. The formazan deposits were dissolved in dimethyl sulfoxide (DMSO) and the absorbance of each well was measured at 540 nm in Multiskan Ascent 96/384 Plate Reader. Each drug concentration was evaluated with six replicates and the experiments were repeated three times.

### 2.8. Western Blot Analysis

Cells were homogenized in RIPA buffer (0.1% SDS, 1% NP40, 0.5% CHAPS), supplemented with protease inhibitors (Sigma-Aldrich/Merck, Darmstadt, Germany)), and were subjected to Western blot analysis as previously described [26]. Briefly, lysates were separated by 4–20% gradient precast SDS-PAGE (Bio-Rad, Hercules, CA, USA) and transferred onto polyvinylidene difluoride (PVDF) membranes (Immobilion P, Millipore, Burlington, MA, USA). Primary antibodies to HER2 (#4290, lot 2), pHER2 (#2247, lot 9), ERK (#4695, lot 14), pERK (#4370, lot 12), Akt (#9272, lot # 24), pAkt (#9271, lot # 13), β-actin (#2247, lot 9) were from Cell Signaling Technology (Danvers, MA, USA) (1:1000). Primary antibody to p-RB (Ser 780) (sc-12901) was from Santa Cruz Biotechnology (Dallas, TX, USA). Secondary antibodies conjugated with peroxidase were anti-rabbit IgG from Sigma-Aldrich/Merck (Darmstadt, Germany) (1:20.000) or anti-mouse IgG from Calbiochem (San Diego, CA, USA) (1:3.000). Membranes were incubated with LiteAblot PLUS reagent (Euroclone, Pero (MI), Italy) and the immunoreactive proteins were detected with ChemiDoc™ XRS-System (Bio-Rad, Hercules, CA, USA).

### 2.9. Flow Cytometry

Sera were collected from immunized mice 14 days after the third vaccination and analyzed by flow cytometry (BD FACSCalibur) using Δ16HER2-HEK293 cells, ectopically expressing on their membrane the Δ16HER2 oncoprotein. In particular, blood was collected from the retro-orbital plexus and allowed to clot at room temperature for 20 min. Serum was separated from blood by two subsequent centrifugations at 2000× *g* at 4 °C. Sub-confluent Δ16HER2-HEK293 cells were detached and dispended at a density of 5 × 10⁵ cells per tube. After centrifugation for 5 min at 129× *g* at 4 °C, the obtained cell pellet was resuspended and washed twice in staining buffer (2% FBS-containing PBS at pH 7.4). Cells were incubated with sera of vaccinated mice (1:40 dilution in staining buffer) for 1 h at 4 °C. MGR2, a murine monoclonal antibody reacting to human HER2 extracellular domain (kindly provided by E. Tagliabue, Department of Experimental Oncology-Istituto Nazionale Tumori, Milano), was used as positive control (10 µg/mL in staining buffer). After incubation, cells were washed three times and incubated on ice in dark with the secondary antibody-FITC (goat anti-mouse IgG H + L, Thermo Fisher Scientific (Waltham, MA, USA), 1:200 dilution in staining buffer). Samples were washed twice and resuspended in 600 µL of staining buffer and read on BD FACSCalibur. Cells Quest pro version (6.0.2) and Flowjo version 8.7 were used as acquisition software and analysis software, respectively.

For antibody-dependent cellular cytotoxicity (ADCC), 1 × 10^4^ CAM6 target cells stained with 2 µM CFSE (Molecular Probes, Eugene, OR, USA) were cultured overnight with splenocytes from untreated wild-type FVB female mice (n = 3), used as effector cells, at different effector:target (E:T) ratios (200:1, 100:1 and 50:1 E:T ratio) in the presence of a 1:50 dilution of sera from vaccinated mice. Cells were then collected and stained with 1 μg/mL 7-Amino-ActinomycinD (7-AAD, BD Biosciences, San Jose, CA, USA), acquired by FACS on a BD FACSVerse and analyzed using Flowjo version 10. ADCC was calculated as described in [31].

### 2.10. Cytotoxicity

Splenocytes (SPC) from vaccinated mice were collected by smashing spleens on a 40 µm pore cell strainer, centrifuging the resulting cells at 300× *g*, for 10 min and incubating them in erythrocyte lysing buffer for 10 min at room temperature. 1 × 10^4^ CAM3 target cells were incubated with 2 µM CFSE (Molecular Probes (Eugene, OR, USA), cod. C34554) for 20 min at 37 °C, washed with RPMI-1640 supplemented with 10% FBS, and then cultured with thawed SPC at different effector:target (E:T) ratios for 48 h. Cells were then stained with 1 μg/mL 7-Amino-ActinomycinD (7-AAD, BD Biosciences (San Jose, CA, USA)), acquired using a BD FACSVerse (BD Biosciences) and analyzed using FlowJO10.5.3. Percent killing was obtained by back-gating on the CFSE^+^ targets and measuring the % of 7-AAD+ dead cells. Percent specific lysis was calculated with the formula [(dead targets in sample (%) − spontaneously dead targets (%))/(dead target maximum-spontaneously dead targets (%))] × 100. Spontaneous release was obtained by culturing target cells without SPC, whereas maximal release was obtained after treatment with PBS supplemented with 1% saponin [31].

### 2.11. Cell Cycle Analysis

BT-474.R cells were seeded in 6-well plates (2 × 10^5^ cells/well) and incubated with immune-IgG antibodies for 72 h. Cells were fixed for 1 h by adding ice-cold 70% ethanol and then washed with staining buffer (PBS, 2% FBS and 0.01% NaN_3_). The cells were treated with 100 μg/mL ribonuclease A solution (Sigma Aldrich/Merck, Darmstadt, Germany), incubated for 30 min at 37 °C, stained for 30 min at room temperature with propidium iodide (PI) 20 μg/mL (Sigma Aldrich/Merck, Darmstadt, Germany) and analyzed on a FACScan flow cytometer (BD FACSCalibur) using BD CellQuest software (Becton Dickinson and company, Franklin Lakes, NJ, USA).

### 2.12. Histological and Immunohistochemical Analysis

Tumor samples were fixed in 10% neutral buffered formalin and embedded into paraffin; slides were cut and stained with Hematoxylin (Bio-Optica, Milano, Italy) and Eosin (Bio-Optica, Milano, Italy) for histological examination. For immunohistochemistry, slides were deparaffinized, serially rehydrated and, after the appropriate antigen retrieval procedure (microwave-sodium citrate buffer, pH 6.0, 10 min), stained with the following primary antibodies: mouse monoclonal anti-PCNA antibody (M0879, Dako (Glostrup, Denmark), 1:800), rabbit p44/42 MAPK (Erk1/2) antibody (#4695, Cell Signaling Technology (Danvers, MA, USA), 1:250) and rabbit Phospho-p44/42 MAPK (Erk1/2) antibody (#4370, Cell Signaling Technology (Danvers, MA, USA), 1:500), followed by the appropriate secondary antibodies (Jackson Laboratories (Bar Harbor, ME, USA), 1:500). Immunoreactive antigens were detected using streptavidin peroxidase (Thermo Fisher Scientific, Waltham, MA, USA) and the DAB Chromogen System (Dako, Glostrup, Denmark) or MACH3 polymer (Biocare Medical, Pacheco, CA, USA) and AEC Chromogen (Biocare Medical, Pacheco, CA, USA). After chromogen incubation, slides were counterstained in Hematoxylin (Bio-Optica, Milano, Italy) and images were acquired by Leica DMRD optical microscope (Leica, Wetzlar, Germany). p-ERK and ERK quantification (% of positive cells) was performed on whole tumors scanned with Nanozoomer scanner from Hamamatsu and analyzed with Qu-Path 0.3.2 software using Pixel classification Tool.

### 2.13. Statistical Analyses

Statistical analysis was carried out with GraphPad Prism 8 Software (San Diego, CA, USA), using the most appropriate test, as specified in each figure. *p* < 0.05 was used as the critical level of significance.

## 3. Results

### 3.1. Vaccination with Anti-HER2 Phage-Based Vaccines Controls BC Growth in ∆16HER2 Mice

**Figure 1:**
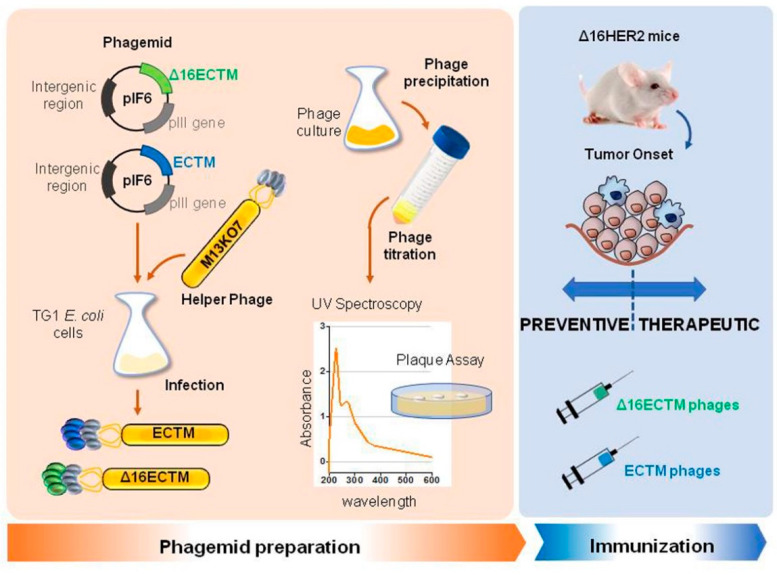
Schematic representation of anti-HER2 phage-based vaccine production.

M13 bacteriophages were engineered to display the EC and TM domains of human HER2 (ECTM phages) or its splice variant Δ16HER2 (Δ16ECTM phages) on their surface as fusion proteins with the coat protein pIII. ECTM or Δ16ECTM phages were released by TG1 bacterial cells, after transformation with recombinant phagemid vector pIF6 followed by IPTG induction and super-infection with M13K07 helper phages. Then, phages were purified and quantified by colony forming assay before being used as anti-HER2 vaccines in ∆16HER2 transgenic mice (Figure 1).

**Figure 2:**
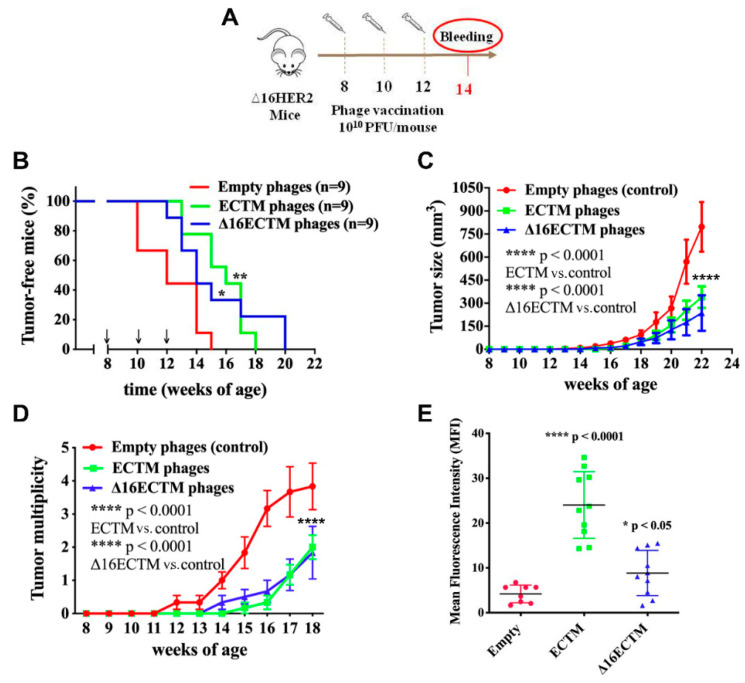
Phage-based vaccination against HER2+ BC in preventive experiment. (**A**). 10^10^ (PFU/mouse) ECTM- or Δ16ECTM- or empty-phage (control) vaccines were administered to ∆16HER2 mice at 8, 10 and 12 weeks of age, prior to tumor onset (n = 9 mice per group). Bleeding occurred at 14th week of age for sera analysis of anti-HER2-antibody titer. (**B**). Kaplan–Meier curves; Log Rank test (** *p* < 0.01 ECTM vs. empty phages; * *p* < 0.05 Δ16ECTM vs. empty phages). (**C**). Tumor growth curves; Two-way ANOVA with Tukey’s multiple comparison test (**** *p* < 0.0001 control vs. ECTM; **** *p* < 0.0001 control vs. Δ16ECTM). (**D**). Tumor multiplicity curves; Two-way ANOVA with Tukey’s multiple comparison test (**** *p* < 0.0001: ECTM vs. empty phages; Δ16ECTM vs. empty phages). (**E**). Antibody detection. Sera of mice were analyzed by FACS using Δ16HER2-HEK293 cells. Data represent Mean fluorescence intensity (MFI) ± SD; Student’s *t*-test (**** *p* < 0.0001 control vs. ECTM; * *p* < 0.05 Δ16ECTM vs. control).

The anticancer potential of ECTM or Δ16ECTM phages was first investigated in a preventive setting by administering the phage-based vaccines i.p. (10^10^ CFU/mouse) before the development of mammary tumors at 8, 10 and 12 weeks of age. Two weeks after the third immunization, serum was collected to analyze vaccine-induced anti-HER2 antibodies (Figure 2A). Immunized mice displayed a significant delayed tumor onset (Figure 2B) and developed significantly smaller and fewer masses compared with the control group, as shown by the mammary tumor growth (Figure 2C) and multiplicity (Figure 2D) curves. All females treated with empty phages developed palpable tumors within 15 weeks of age, when 75% of mice vaccinated with ECTM-phages and 40% of those vaccinated with Δ16ECTM phages were still tumor-free (Figure 2B). Moreover, one month after the last vaccine administration, each mouse of the control group had on average three tumors, while vaccinated mice had on average less than one, although at least one palpable tumor arose in each of them within 20 weeks of age (Figure 2B–D). The antitumor protection elicited by both ECTM and Δ16ECTM phages was associated with a significant anti-HER2 antibody production, which resulted higher in ECTM-phages-vaccinated mice, as assessed by flow cytometry (Figure 2E). Δ16ECTM phages were also able to induce T-cell cytotoxicity, as verified in vitro by incubating splenocytes from immunized mice with a Δ16HER2-positive BC cell line (Supplementary Figure S1).

**Figure 3:**
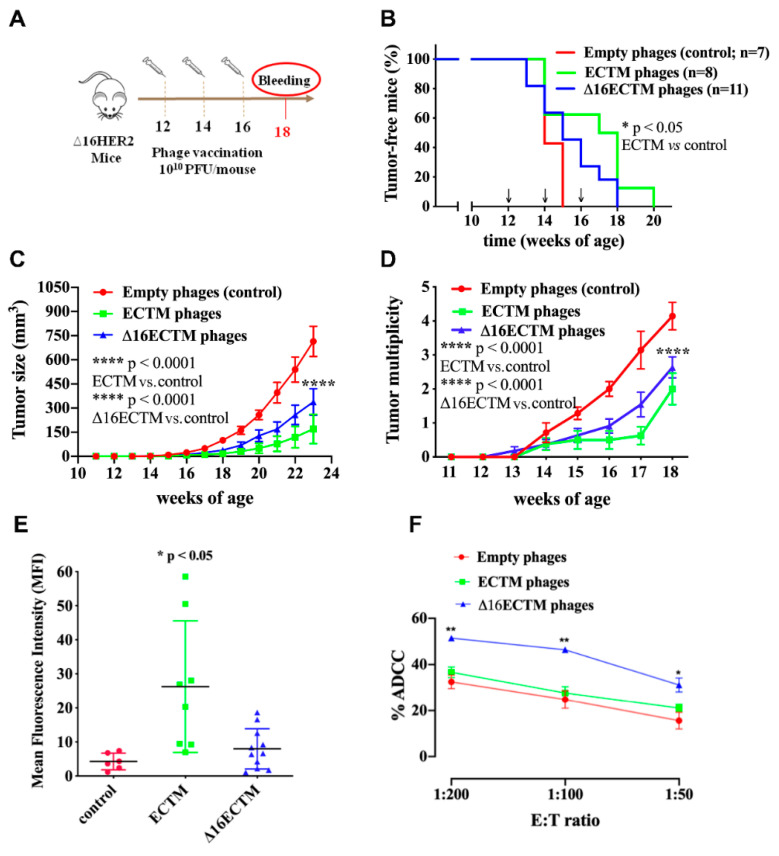
Phage-based vaccination against HER2+ BC in a therapeutic experiment. (**A**). 10^10^ (PFU/mouse) ECTM- or Δ16ECTM- or empty-phage (control) vaccines were administered to ∆16HER2 mice at 12, 14 and 16 weeks of age (*n* = 7–11 mice per group). Bleeding occurred at the 18th week of age for sera analysis of anti-HER2-antibody titer. (**B**). Kaplan–Meier curves; Log Rank test (* *p* < 0.05 ECTM vs. empty phages). (**C**). Tumor growth curves; Two-way ANOVA with Tukey’s multiple comparison test (**** *p* < 0.0001 control vs. ECTM; **** *p* < 0.0001 control vs. Δ16ECTM). (**D**). Tumor multiplicity curves; Two-way ANOVA with Tukey’s multiple comparison test (**** *p* < 0.0001: ECTM vs. empty phages; Δ16ECTM vs. empty phages). (**E**). Antibody detection. Sera of mice (n = 7–11 mice per group) were analyzed by FACS using Δ16HER2-HEK293 cells; data represent Mean fluorescence intensity (MFI) ± SD; Student’s *t*-test (* *p* < 0.05 control vs. ECTM). (**F**). ADCC assay was performed using CAM6 target cells incubated with a 1:50 dilution of sera from vaccinated mice (n = 3) and splenocytes as effector cells at different effector/target cells ratios (200:1, 100:1 and 50:1). Results shown are the mean ± SEM of the percentage of ADCC induced by different sera; Student’s *t*-test (* *p* < 0.05; ** *p* < 0.01).

To evaluate the therapeutic efficacy of ECTM and Δ16ECTM phage-based vaccines, the immunization schedule was postponed by one month, starting the treatments of Δ16HER2 transgenic females at 12 weeks of age, when the process of carcinogenesis was already initiated (Figure 3A). Indeed, at this time whole-mount analyses showed neoplastic lesions in the mammary glands, even in mice that had not developed palpable tumors yet [28,32]. As shown in Figure 3B, vaccination with ECTM-phages resulted in a statistically significant delay in tumor onset, with 60% of mice completely protected at 15 weeks of age, when all control mice had at least one palpable tumor. Δ16ECTM-phages were also able to prolong tumor latency with respect to control, although with no statistically significant differences (Figure 3B). Both ECTM- and Δ16ECTM-phage vaccines were able to significantly reduce tumor growth rate (Figure 3C) and multiplicity (Figure 3D), and to induce antibodies toward HER2, in correlation with the conferred antitumor protection (Figure 3E). ECTM-phages elicited a statistically significant higher anti-HER2 antibody titer with respect to control, even if a high variability was observed within this experimental group. Of note, Δ16ECTM phages induced a lower anti-HER2 antibody response in comparison with ECTM-phages, but the Δ16ECTM-phage-induced antibodies can contribute to protection by mediating ADCC, as demonstrated ex vivo using the CAM6 cell line (Figure 3F), which can be considered the in vitro counterpart of Δ16HER2 mammary tumors [29].

**Figure 4:**
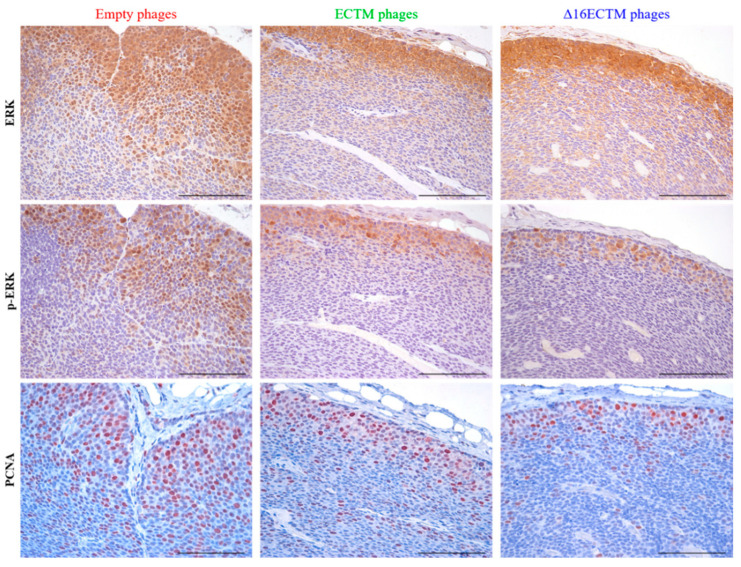
Ex vivo antitumor effects induced by phage-based vaccinations. Representative immunohistochemical images of tumors (n = 3 tumors from 3 mice per group) excised at the end of the therapeutic experiment, stained with ERK, phospho-ERK and PCNA antibodies (brown cells). Scale bar 100 µm. Quantitative analysis of p-ERK+/ERK+ cells is shown in Supplementary Figure S2.

Evaluation of ERK and phospho-ERK by immunohistochemistry indicated a strong cytoplasmatic and nuclear staining at the invasion front of tumors treated with empty phages and a downregulation of both markers in tumors treated with both ECTM- and Δ16ECTM-phages, suggesting that elevated MAPK activity, evident in control tumors, was strongly inhibited by both vaccines in treated tumors (Figure 4). The reduced p-ERK signal after vaccination with ECTM- and Δ16ECTM-phages was confirmed by the reduced ratio of p-ERK+/ERK+ cells (Supplementary Figure S2). Immunohistochemical analysis of tumors also revealed a significant reduction in the percentage of proliferating cell nuclear antigen (PCNA)-positive cells after treatments, confirming that anti-HER2 phage-based vaccination is able to reduce BC cell proliferation (Figure 4).

### 3.2. In Vitro Analysis of Anticancer Effects of Anti-HER2 Antibodies Elicited by Phage-Based Vaccines

**Figure 5:**
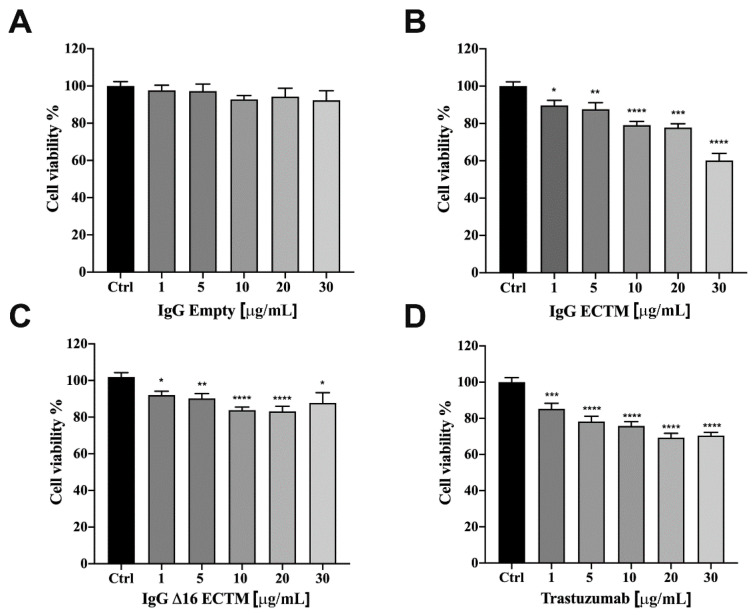
IgG purified from ECTM- and Δ16ECTM-immune sera decreased cell viability in trastuzumab sensitive-BT-474 BC cells. Cells were incubated for 72 h in the presence of increasing concentrations of control IgG (**A**), ECTM-IgG (**B**), Δ16ECTM-IgG (**C**) or trastuzumab (**D**) and cell viability was determined by MTT assay. The results are expressed as percentage of living cells ± SEM with respect to control (untreated cells). Columns show the means of results obtained in three separate experiments, wherein each treatment was repeated in 6 wells. One-way ANOVA followed by Dunnett’s post-hoc tests (* *p* < 0.05; ** *p* < 0.01; *** *p* < 0.001; **** *p* < 0.0001 vs. control).

The anticancer activity of vaccine-elicited anti-HER2 antibodies was analyzed in vitro by MTT assay, first on murine CAM6 cells, then on trastuzumab-sensitive BT-474 human BC cells. IgG antibodies (from 1 to 30 μg/mL) purified from sera of mice vaccinated with ECTM and Δ16ECTM phages reduced CAM6 cell viability after 72 h of incubation (Supplementary Figure S3). A similar anticancer effect mediated by ECTM- and Δ16ECTM-IgG antibodies was observed using human BT-474 BC cells as the target. Indeed, control IgG antibodies were not effective, but ECTM-IgG antibodies decreased BT-474 cell viability in a dose-dependent manner after 72 h of incubation (Figure 5A,B) and showed a comparable efficacy to trastuzumab, resulting in a 40 and 30% reduction in cell viability, respectively, when administered at 30 μg/mL (Figure 5B,D). Δ16ECTM-IgG also resulted in an inhibition of BT-474 cell viability, although never exceeding 20% (Figure 5C).

**Figure 6:**
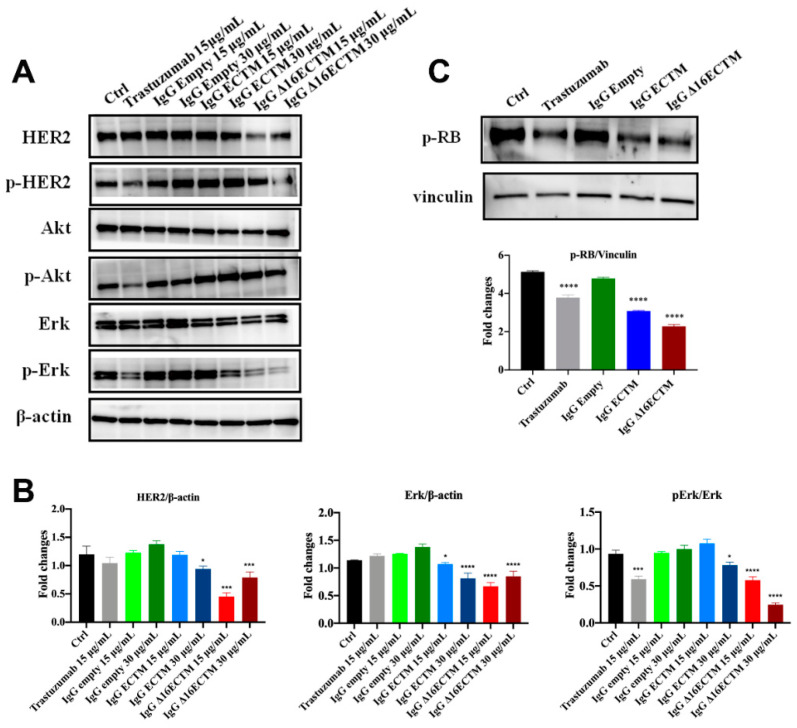
IgG purified from ECTM- and Δ16ECTM-immune sera impaired ERK activation in BT-474 BC cells. (**A**). Representative Western blot analysis of HER2 downstream signaling pathways in BT-474 cells, treated or not with trastuzumab or control-IgG or immune-IgG at the indicated concentrations for 8 h. Expression level of HER2, pHER2, AKT, pAKT, ERK, pERK was analyzed. Equal amounts of protein (20 μg) were loaded and β-actin was used as loading control. Data are representative of a typical experiment repeated three times with similar results. (**B**). Densitometric quantification of HER2 and ERK expression, normalized on β-actin, and of pERK/ERK from three independent experiments were shown; one-way ANOVA test followed by Tukey’s multiple comparison test (* *p* ≤ 0.05; *** *p* ≤ 0.001; **** *p* ≤ 0.0001: IgG ECTM or IgG Δ16ECTM vs. IgG empty; trastuzumab vs. control). (**C**). IgG purified from ECTM- and Δ16ECTM-immune sera reduced the levels of phosphorylated RB in BT-474 cells. Upper panel: representative Western blot analysis of phosphorylated RB (Ser 780) in BT-474 cells, treated or not with 15 μg/mL trastuzumab or control-IgG or immune-IgG (30 μg/mL) for 8 h. Equal amounts of protein (20 μg) were loaded and vinculin was used as a loading control. Data are representative of a typical experiment repeated three times with similar results. Lower panel: densitometric quantification of phosphorylated RB expression, normalized on vinculin, was shown; one-way ANOVA test followed by Tukey’s multiple comparison test **** *p* ≤ 0.0001: IgG ECTM or IgG Δ16ECTM vs. IgG empty; trastuzumab vs. control. The original western blot data can be found in Supplementary Materials File S1 (pages 1–3).

The molecular mechanisms underlying the anticancer effect of vaccine-elicited anti-HER2 antibodies were analyzed by Western blot on BT-474 cells (Figure 6). In particular, we investigated whether anti-HER2 antibodies induced by phage-based vaccination were able to impact on HER2, AKT and ERK expression level and phosphorylation status by treating BT-474 cells with control or immune IgG antibodies (15 and 30 μg/mL) or trastuzumab. As expected, trastuzumab downregulated HER2 activation and consequently reduced AKT and ERK phosphorylation (Figure 6A,B). Interestingly, both ECTM- and Δ16ECTM-IgG treatments induced a marked downregulation of ERK expression and activation with respect to control IgG antibodies, confirming the results obtained in vivo (Figure 4), whereas phosphorylated AKT levels were not decreased in IgG-treated cells (Figure 6A,B). A clear reduction in HER2 expression was observed when BT-474 cells were incubated with Δ16ECTM-IgG (Figure 6A,B).

**Figure 7:**
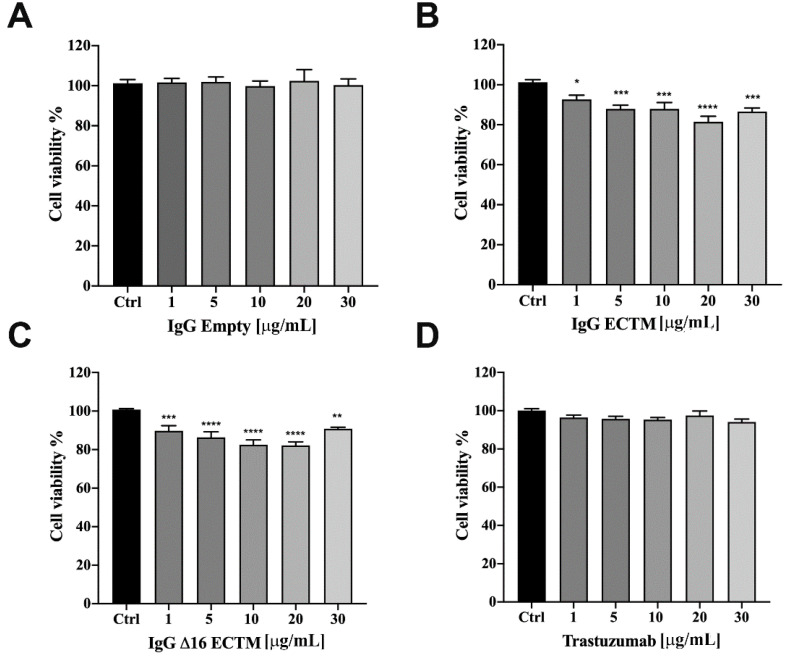
IgG purified from ECTM- and Δ16ECTM-immune sera decreased cell viability in trastuzumab-resistant BT-474.R BC cells. Cells were incubated for 72 h in the presence of increasing concentrations of control IgG (**A**) or ECTM-IgG (**B**) or Δ16ECTM-IgG (**C**) or trastuzumab (**D**) and cell viability was determined by MTT assay. The results are expressed as percentage of living cells ± SEM with respect to control (untreated cells). Columns report the means of mean of three separate experiments, wherein each treatment was repeated in 6 wells. One-way ANOVA followed by Dunnett’s post-hoc tests (* *p* < 0.05; ** *p* < 0.01; *** *p* < 0.001; **** *p* < 0.0001 vs. control).

Considering the key role of retinoblastoma (RB) phosphorylation in the regulation of cell cycle progression downstream of the MAPK pathway [33,34], we performed a Western blot to detect the phosphorylation status of RB in BT-474 cells treated or not with trastuzumab, ECTM- and Δ16ECTM-IgG. As shown in Figure 6C, RB protein was hyperphosphorylated (Ser 780) in untreated BT-474 cells and in BT-474 cells treated with control IgG, leading to cancer cell proliferation. By contrast, ECTM- and Δ16ECTM-IgG as well as trastuzumab were effective at reactivating RB function by decreasing protein phosphorylation in this BC cell line, likely reducing its proliferation. After assessing the anticancer effects of anti-HER2 antibodies elicited by phage-based vaccines in BT-474 cells, ECTM- and Δ16ECTM-IgG were also tested on their trastuzumab-resistant counterpart. Establishment of trastuzumab resistance was confirmed by a cell viability assay showing that trastuzumab-resistant BT-474.R cell proliferation was only minimally affected by trastuzumab treatment, ranging from 1 to 75 μg/mL, while the viability of trastuzumab-sensitive BT-474 cells significantly decreased, reaching 40% of inhibition at the highest trastuzumab concentration (Supplementary Figure S4A). Accordingly, Western blot analysis revealed that 15 μg/mL trastuzumab inhibited in a time-dependent manner the PI3K/AKT and MAPK/ERK signaling pathways in BT-474 cells, whereas it was not able to counteract the overactivation of HER2 downstream pathways in BT-474.R cells, displaying even an increased level of pHER2/HER2 and pERK/ERK upon trastuzumab re-exposure due to the acquisition of trastuzumab resistance (Supplementary Figure S4B), in agreement with previous studies [35]. Treatment of trastuzumab-resistant BT-474.R cells with ECTM- or Δ16ECTM-IgG for 72 h resulted in a dose-dependent reduction in cell viability, reaching a plateau at 30 μg/mL, whereas trastuzumab was totally ineffective, as expected, as well as control IgG antibodies (Figure 7).

**Figure 8:**
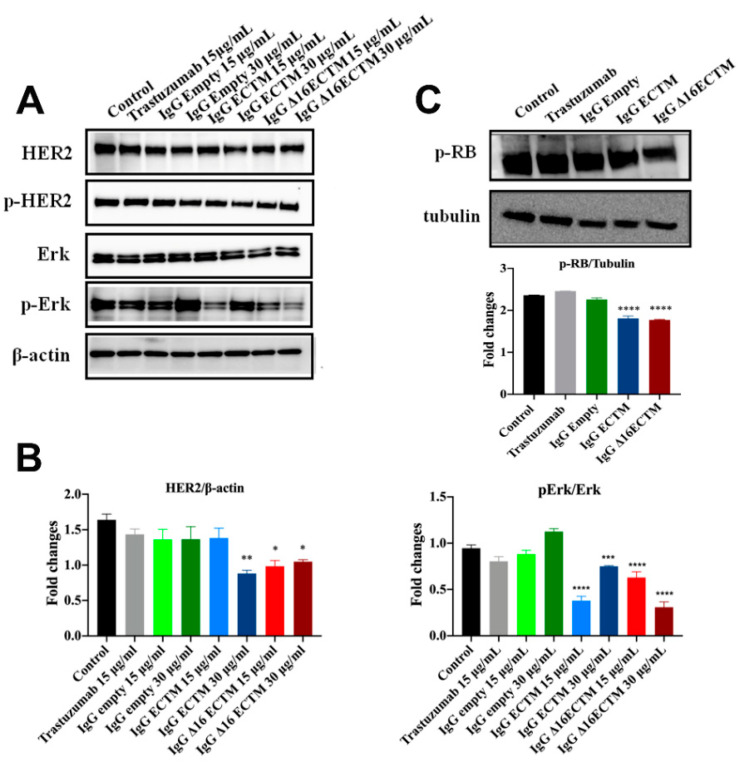
IgG purified from ECTM- and Δ16ECTM-immune sera impaired ERK activation in trastuzumab-resistant BT-474 BC cells. (**A**). Representative Western blot analysis of HER2 downstream signaling pathways in trastuzumab-resistant BT-474 cells, treated or not with trastuzumab or control-IgG or immune-IgG at the indicated concentrations for 8 h. Expression level of HER2, pHER2, ERK, pERK was analyzed. Equal amounts of protein (20 μg) were loaded and β-actin was used as loading control. Data are representative of a typical experiment repeated three times with similar results. (**B**). Densitometric quantification of HER2 and ERK expression, normalized on β-actin, and of pERK/ERK from three independent experiments were shown; one-way ANOVA test followed by Tukey’s multiple comparison test (* *p* ≤ 0.05; ** *p* ≤ 0.01; *** *p* ≤ 0.001; **** *p* ≤ 0.0001: IgG ECTM or IgG Δ16ECTM vs. IgG empty/control). (**C**). IgG purified from ECTM- and Δ16ECTM-immune sera reduced the levels of phosphorylated RB in trastuzumab-resistant BT-474.R BC cells. Upper panel: representative Western blot analysis of phosphorylated RB (Ser 780) in trastuzumab-resistant BT-474.R BC cells, treated or not with 15 μg/mL trastuzumab or control-IgG or immune-IgG (30 μg/mL) for 8 h. Equal amounts of protein (20 μg) were loaded and tubulin was used as loading control. Data are representative of a typical experiment repeated three times with similar results. Lower panel: densitometric quantification of phosphorylated RB expression, normalized on tubulin, was shown; one-way ANOVA test followed by Tukey’s multiple comparison test **** *p* ≤ 0.0001: IgG ECTM or IgG Δ16ECTM vs. IgG empty. The original western blot data can be found in Supplementary Materials File S1 (pages 4–6).

Considering the unique ability of both ECTM- and Δ16ECTM-IgG treatments to impair ERK activation, we evaluated their effect on HER2 downstream signaling pathways in trastuzumab-resistant BT-474.R cells by Western blot, focusing on ERK expression and phosphorylation. In this experimental model, trastuzumab was able to interfere neither with HER2 expression and activation nor with HER2-downstream signaling pathways, as expected. By contrast, a downregulation of HER2 associated with a significant reduction in ERK phosphorylation was observed when trastuzumab-resistant BT-474.R cells were incubated for 8 h with ECTM- or Δ16ECTM-IgG with respect to control IgG-treated cells (Figure 8A,B). Thus, anti-HER2 IgG antibodies elicited by phage-vaccination were able to counteract the sustained activation of oncogenic signaling pathways that characterize trastuzumab-resistant BT-474.R BC cells. In agreement with these results, RB protein was hyperphosphorylated (Ser 780) in untreated and trastuzumab treated BT-474.R cells as well as in cells incubated with control IgG. Interestingly, ECTM- and Δ16ECTM-IgG antibodies were able to rescue RB function by reducing protein phosphorylation (Figure 8C). Consequently, ECTM- and Δ16ECTM-IgG antibodies promoted an accumulation of BT-474.R cells in the G_1_ phase of the cell cycle in comparison with the control group (Supplementary Table S1).

## 4. Discussion

The ability of the immune system to detect and eliminate cancer cells has been widely recognized and the last decade has seen a renaissance of anticancer immunotherapies. The generation of immunological memory and minimal toxicity represent the main advantages of cancer vaccines. However, results from therapeutic BC vaccine clinical trials conducted so far have not been satisfactory due to different reasons, including advanced-stage cancer, heavily pre-treated patients, cancer immune-evasive mechanisms, immunological tolerance against the target antigen and the chosen vaccine platform [36,37,38]. Bacteriophage-based vaccines are potentially able to overcome the limitations of classical vaccines, thanks to their inherent properties. Filamentous phages are capable of achieving an effective antigen presentation to immune cells, to improve the stability and immunogenicity of displayed antigens and to break immune tolerance against self-antigens, as it is the HER2 [23,39]. In this study, we demonstrated that anti-HER2 vaccination using the M13 bacteriophage platform induces a significant anti-HER2 antibody response and controls the tumor growth in a BC preclinical model tolerant to human ∆16HER2. Both ECTM and Δ16ECTM phages were effective not only when administered before cancer development, confirming previously reported results [23], but even in a therapeutic setting. ECTM phages were able to induce a higher anti-HER2 antibody titer than Δ16ECTM phages, in agreement with the elicited protection. Since ∆16HER2 transgenic mice express the transgene in the thymus, ∆16HER2 is a self-antigen due to central tolerance [21]. Although Δ16HER2 differs from wild-type HER2 only because it lacks 16 amino acid residues (amino acids 634 to 649) in the juxtamembrane region, ECTM phages might be able to break immunological tolerance more efficiently than ∆16HER2 phages inducing a stronger antibody response and consequently a higher effectiveness. Of note, despite the lower antibody titer, Δ16ECTM-phage-induced antibodies can better mediate ADCC, contributing to antitumor protection. ADCC is a cell-mediated immune response by which immune cells, typically Natural Killer (NK) cells, cause cell death when specific antibodies are bound to the target cell membrane, and it is considered one of the main mechanisms underlying the therapeutic effect of various monoclonal antibodies used as cancer therapies [40]. The unique ability of Δ16ECTM antibodies to trigger ADCC might be explained considering their higher specificity for the target antigen exposed on CAM6 cancer cells. Indeed, it has been demonstrated that antibody specificity plays a fundamental role in the regulation of Fc-dependent effector functions such as ADCC [41]. The functionality of HER2 immune serum was measured in vitro on BT-474 human BC cells, which are characterized by the co-expression of both HER2 and Δ16HER2 isoform [25,32]. ECTM- and Δ16ECTM-IgG antibodies purified from immune sera inhibited cancer cell viability mainly by interfering with ERK activation and leading to the reactivation of RB in both trastuzumab-sensitive and -resistant BT-474 cells. Consistently, Montgomery et al. reported that antibodies induced by HER2 peptide vaccination in patients are capable of inhibiting HER2 phosphorylation and downstream activation of ERK leading to the subsequent suppression of cancer cell proliferation [42]. Although other signaling pathways are clearly important in HER2-mediated transformation, activation of ERK is generally considered the principal driver of cell proliferation. The MAPK/ERK signaling pathway can control cell cycle progression from G1 to S phase through the induction of cyclin D expression and consequent retinoblastoma (RB) phosphorylation. The hyperphosphorylation of RB protein results in its inactivation, causes the release of the transcription factor E2F and thus leads to cell proliferation. The RB protein is inactivated by phosphorylation in many human cancers [33,35]. Intriguingly, a switch in pathway dependence has been observed from PI3K/AKT to MEK/ERK in advanced HER2+ BC, leading to resistance to anti-HER2 therapies, and making this type of cancer sensitive to MEK/ERK inhibitors [43]. The superior performance of polyclonal anti-HER2 antibodies induced by phage vaccination with respect to the monoclonal antibody trastuzumab might be due to their higher ability to cause HER2 internalization associated with reduction in HER2 signaling. Our finding is consistent with a previous study showing that polyclonal anti-HER2 antibodies induced by an adenovirus-based vaccine against human HER2 were remarkably more potent than trastuzumab in causing HER2 internalization and degradation [44]. This different ability can be particularly important for Δ16HER2, since it is known that this isoform displays an altered internalization, which affects response to therapy and may confer resistance to therapeutic T-DM1 antibody-drug conjugate [45]. The influence of Δ16HER2 on the response to different anti-HER2 antibodies and trastuzumab can be even more relevant in BT-474.R cells, displaying an increase in the ratio of Δ16HER2 over wild-type-HER2 compared to that in parental BT474 cells [46].

## 5. Conclusions

BC is the most common female cancer. HER2+ BC accounts for almost 30% of all breast cancer cases and is associated with poor prognosis. HER2+ breast cancer patients would greatly benefit from therapeutic vaccines, but no vaccines are available yet. The difficulty of breaking immune tolerance represents a major obstacle in tumor vaccine technology. In the present work, we demonstrated that anti-HER2 vaccination using the M13 bacteriophage platform induces a significant anti-HER2 antibody response and controls the tumor growth in a breast cancer preclinical model tolerant to human HER2 self-antigen. Overall, our study provides evidence that anti-HER2 phage-based vaccines represent a safe and successful immunotherapeutic strategy to elicit a protective immunity and to prevent relapse in HER2+ BC patients, particularly for those who develop resistance to trastuzumab.

## Data Availability

The data presented in this study are available in this article and supplementary material.

## Supplementary Materials

The following supporting information can be downloaded at: https://www.mdpi.com/article/10.3390/cancers14164054/s1, Figure S1: Phage-based vaccination against HER2 induced T-cell cytotoxicity. Figure S2: Phage-based vaccination against HER2 reduced p-ERK signal. Figure S3: IgG purified from ECTM- and Δ16ECTM-immune sera decreased cell viability in murine CAM6 cells; Figure S4: Establishment of BT-474 trastuzumab-resistant BC cell line (acquired resistance). Table S1: low cytometry cell cycle analysis of BT-474.R cells treated with ECTM- or Δ16ECTM-IgG antibodies for 72 h. File S1: The original western blot data.
