# Supplementary material for: HER2-Displaying M13 Bacteriophages induce Therapeutic Immunity against Breast Cancer
Source: Cancers (Basel). 2022 Aug 22;14(16):4054. doi: 10.3390/cancers14164054 (PMC9406369; doi:10.3390/cancers14164054)
Supplement: Supplementary file 1 [file cancers-14-04054-s001.zip › cancers-1867438-supplementary.pdf]

Supplementary Material

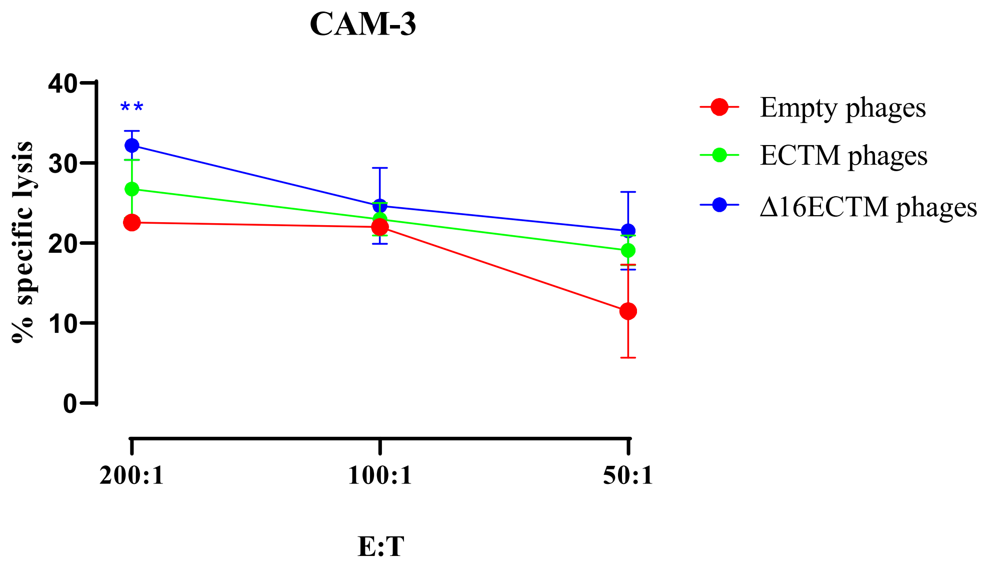

Supplementary Figure S1. Phage-based vaccination against HER2 induced T-cell cytotoxicity. Cytotoxic assay was performed using Δ16HER2-positive CAM3 target (T) cells incubated with splenocytes collected from vaccinated mice (n=3 mice/ group) as effector (E) cells at different effector/target cell ratios (E:T 200:1, 100:1, and 50:1). Results show the mean ± SEM of the percentage of 7-AAD+ dead cells among CFSE+ CAM3 cells (specific lysis), after 48 hours of co-culture with splenocytes. Student's *t*-test (**p = 0.082).

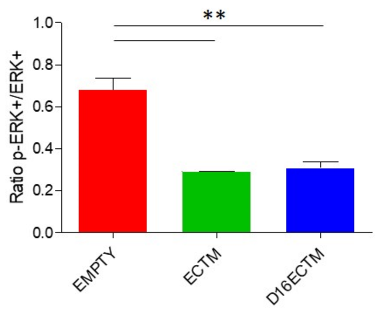

Supplementary Figure S2. Phage-based vaccination against HER2 reduced p-ERK signal. Ratio of p-ERK+/ERK+ cells was performed quantifying the percentage of p-ERK and ERK positive cells on whole tumors (n=3 tumors from 3 mice per group), scanned with Nanozoomer scanner from Hamamatsu and analyzed with Qu-Path 0.3.2 software using Pixel classification Tool. Results show the mean ± SEM. Student's *t*-test (**p < 0.0031).

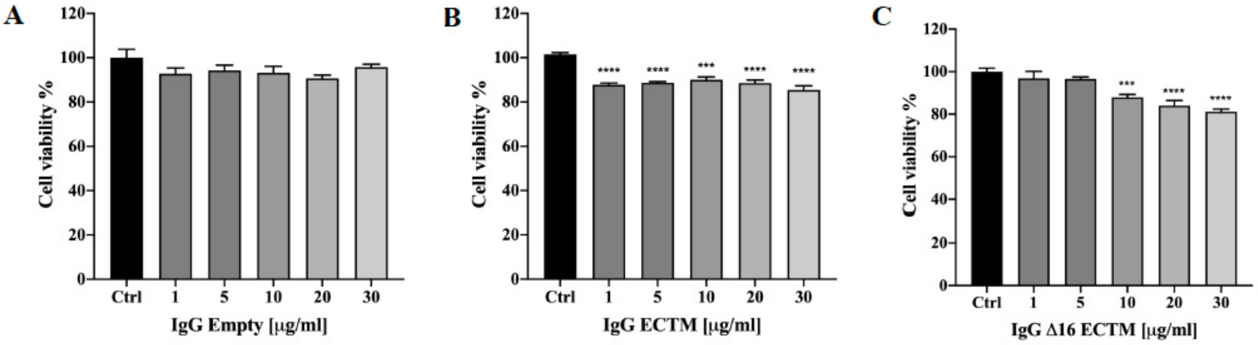

Supplementary Figure S3. IgG purified from ECTM- and Δ16ECTM-immune sera decreased cell viability in murine CAM6 cells. Cells were incubated for 72 hours in the presence of increasing concentrations of control IgG (A) or ECTM-IgG (B) or Δ16ECTM-IgG (C) and cell viability was determined by MTT assay. The results are expressed as percentage of living cells ± SEM with respect to control (untreated cells). Columns represent the means of three separate experiments, wherein each treatment was repeated in 6 wells. One-way ANOVA followed by Dunnett's post-hoc tests (*** p < 0.001; **** p < 0.0001 vs. control).

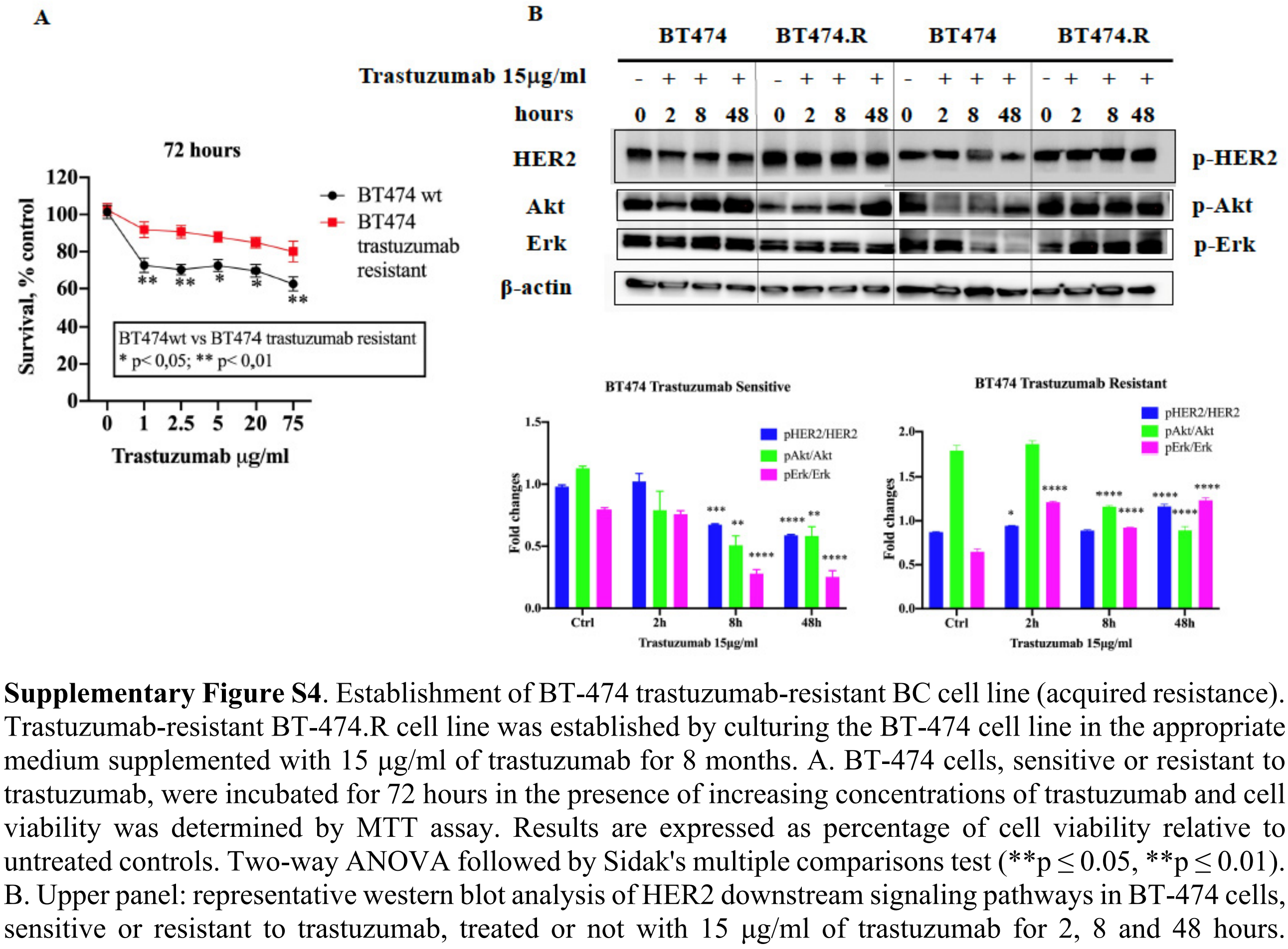

Supplementary Figure S4. Establishment of BT-474 trastuzumab-resistant BC cell line (acquired resistance). Trastuzumab-resistant BT-474.R cell line was established by culturing the BT-474 cell line in the appropriate medium supplemented with 15 µg/ml of trastuzumab for 8 months. A. BT-474 cells, sensitive or resistant to trastuzumab, were incubated for 72 hours in the presence of increasing concentrations of trastuzumab and cell viability was determined by MTT assay. Results are expressed as percentage of cell viability relative to untreated controls. Two-way ANOVA followed by Sidak's multiple comparisons test (**p ≤ 0.05, **p ≤ 0.01). B. Upper panel: representative western blot analysis of HER2 downstream signaling pathways in BT-474 cells, sensitive or resistant to trastuzumab, treated or not with 15 µg/ml of trastuzumab for 2, 8 and 48 hours. PI3K/AKT and MAPK/ERK signaling pathways were analyzed. Equal amounts of protein (20 µg) were loaded and β-actin was used as loading control. Data are representative of a typical experiment repeated three times with similar results. Lower panels: densitometric quantification of pHER2/HER2, pAKT/AKT, pERK/ERK. One-way ANOVA test followed by Dunnett's multiple comparison test (*p ≤ 0.05; ***p ≤ 0.001; ****p ≤ 0.0001).

Supplementary Table S1.
Flow cytometry cell cycle analysis of BT-474.R cells treated with ECTM- or Δ16ECTM-IgG antibodies for 72 hours.

Treatments	% G1	% S	% G2/M
Control	58 ± 3.5	21.8 ± 5	20.2 ± 3
IgG ECTM 30 μg/mL	68.6 ± 3.4 [a]	9.4 ± 3.5 [c]	19 ± 1.5
IgG Δ16ECTM 30 μg/mL	65.8 ± 2.5 [b]	7.2 ± 3.5 [c]	19.6 ± 1.5

Values are mean ± SD n=5
One-way ANOVA test followed by Dunnett's multiple comparison test: a ****$p \leq 0.0001$; b ***$p \leq 0.001$; c **$p \leq 0.01$
