# Supplementary material for: HER2-Displaying M13 Bacteriophages induce Therapeutic Immunity against Breast Cancer
Source: Cancers (Basel). 2022 Aug 22;14(16):4054. doi: 10.3390/cancers14164054 (PMC9406369; doi:10.3390/cancers14164054)
Supplement: Supplementary file 1 [file cancers-14-04054-s001.zip › Supplementary Materials File S1.pdf]

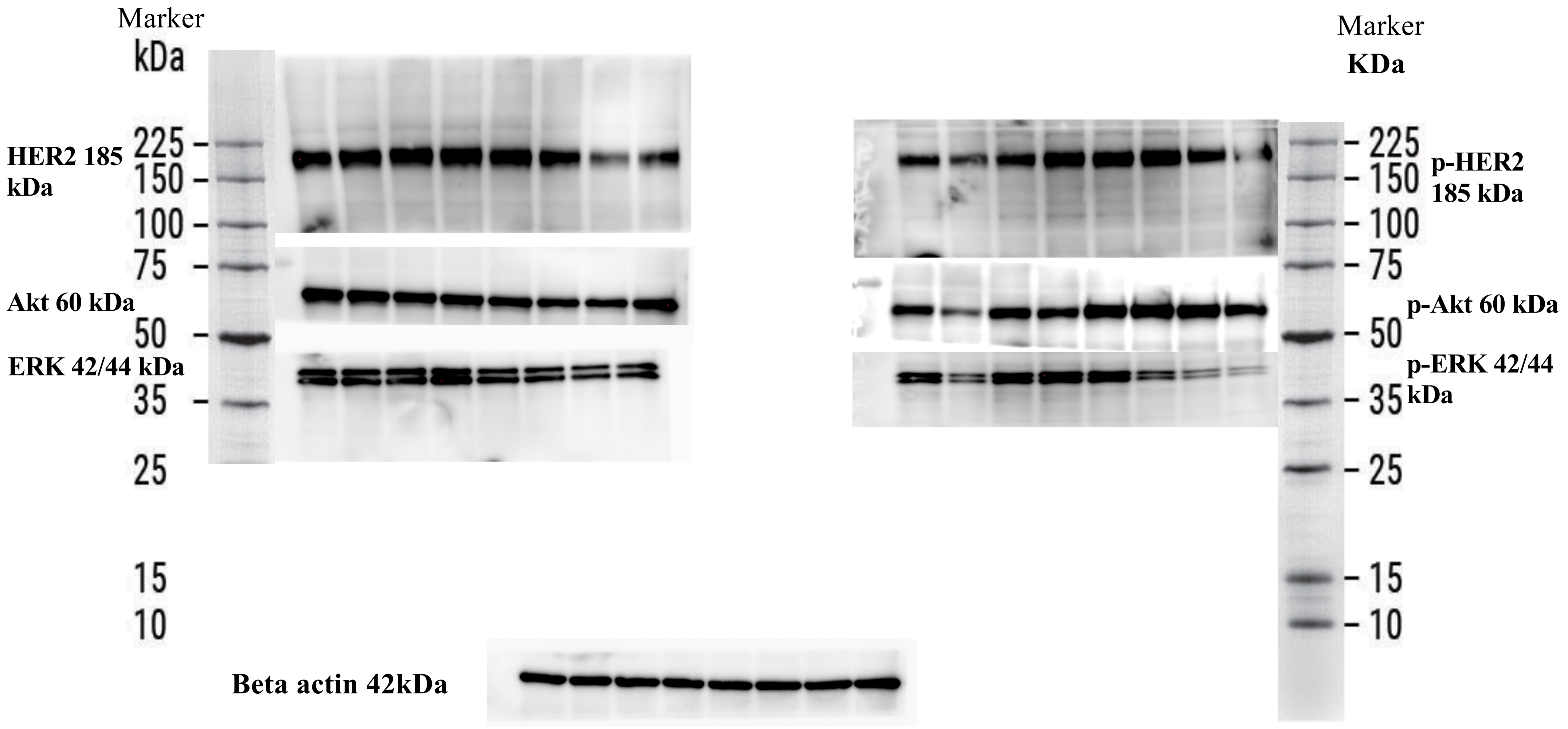

Original western blots from which Figure 6 (Panel A) derived:
analysis of the expression levels of HER2, pHER2, Akt, pAkt, ERK, pERK and β-actin in trastuzumab sensitive BT-474 cells, treated or not with trastuzumab or control-IgG or immune-IgG for 8 hours.

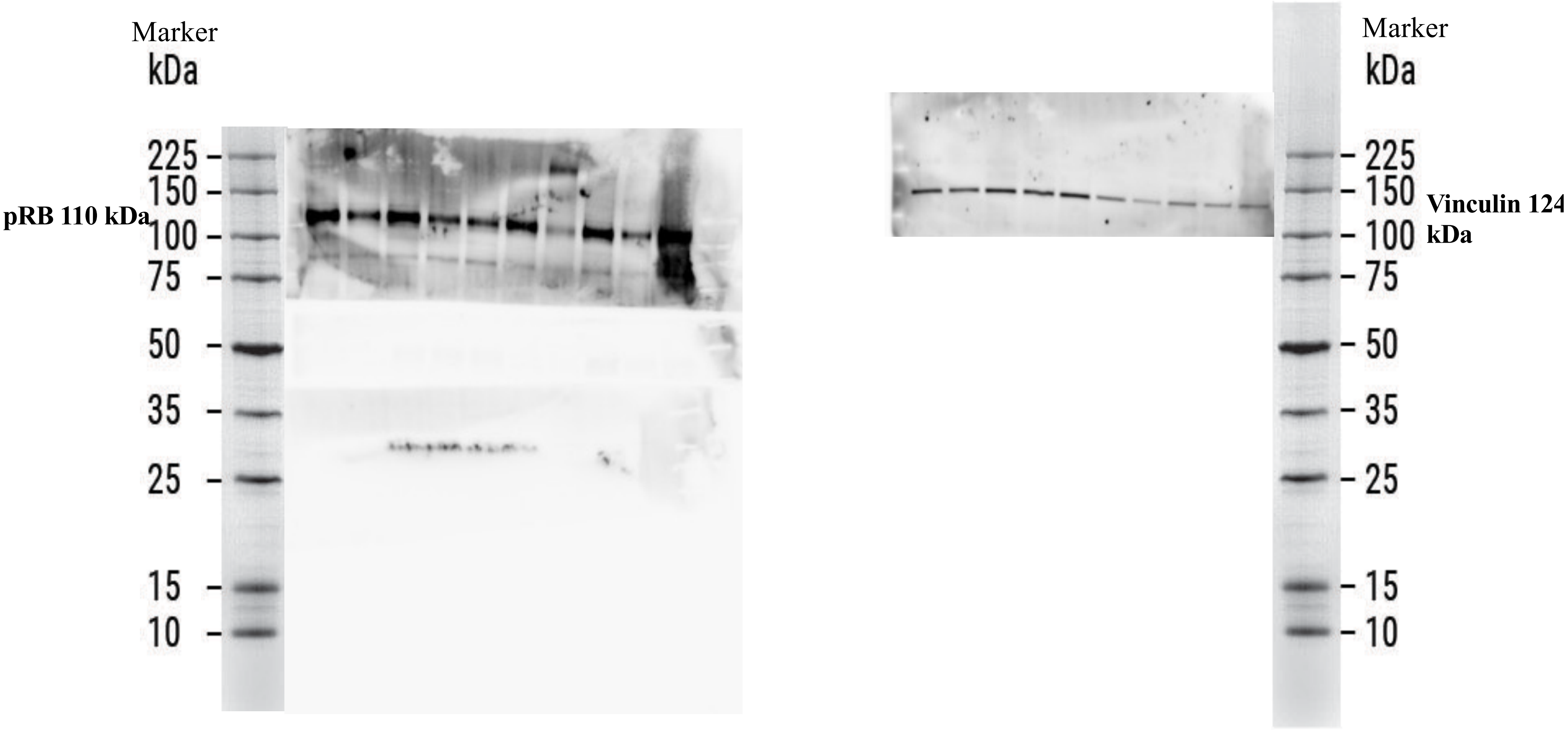

Original western blots from which Figure 6 (Panel C) derived:
analysis of the expression levels of pRB and vinculin in trastuzumab sensitive BT-474 cells, treated or not with trastuzumab or control-IgG or immune-IgG for 8 hours (lanes 1-5: samples shown in the Figure 6).

	HER2/β-actin	ERK/β-actin	pERK/ERK	pAKT/AKT	pRB/Vinculin
Ctrl	1.1619352	1.136381032	0.90888449	0.785939356	5.136666667
Trastuzumab 15μg/ml	0.998749912	1.231663141	0.553886772	0.595614235	3.783333333
IgG empty15μg/ml	1.220824649	1.254265141	0.959528715	0.99745692	-
IgG empty 30 μg/ml	1.366925333	1.383872968	1.035681769	0.896702614	4.786666667
IgG ECTM 15μg/ml	1.258655056	1.090137162	1.082599443	1.005735603	-
IgG ECTM 30 μg/ml	0.953788042	0.812949194	0.775646206	1.370403968	3.089080289
IgG Δ16 ECTM 15μg/ml	0.447996027	0.686202358	0.587672756	1.269266502	-
IgG Δ16 ECTM 30μg/ml	0.791426762	0.78987637	0.252087391	0.707616232	2.283049442

Corresponding to Figure 6. Densitometry Readings/intensity Ratio

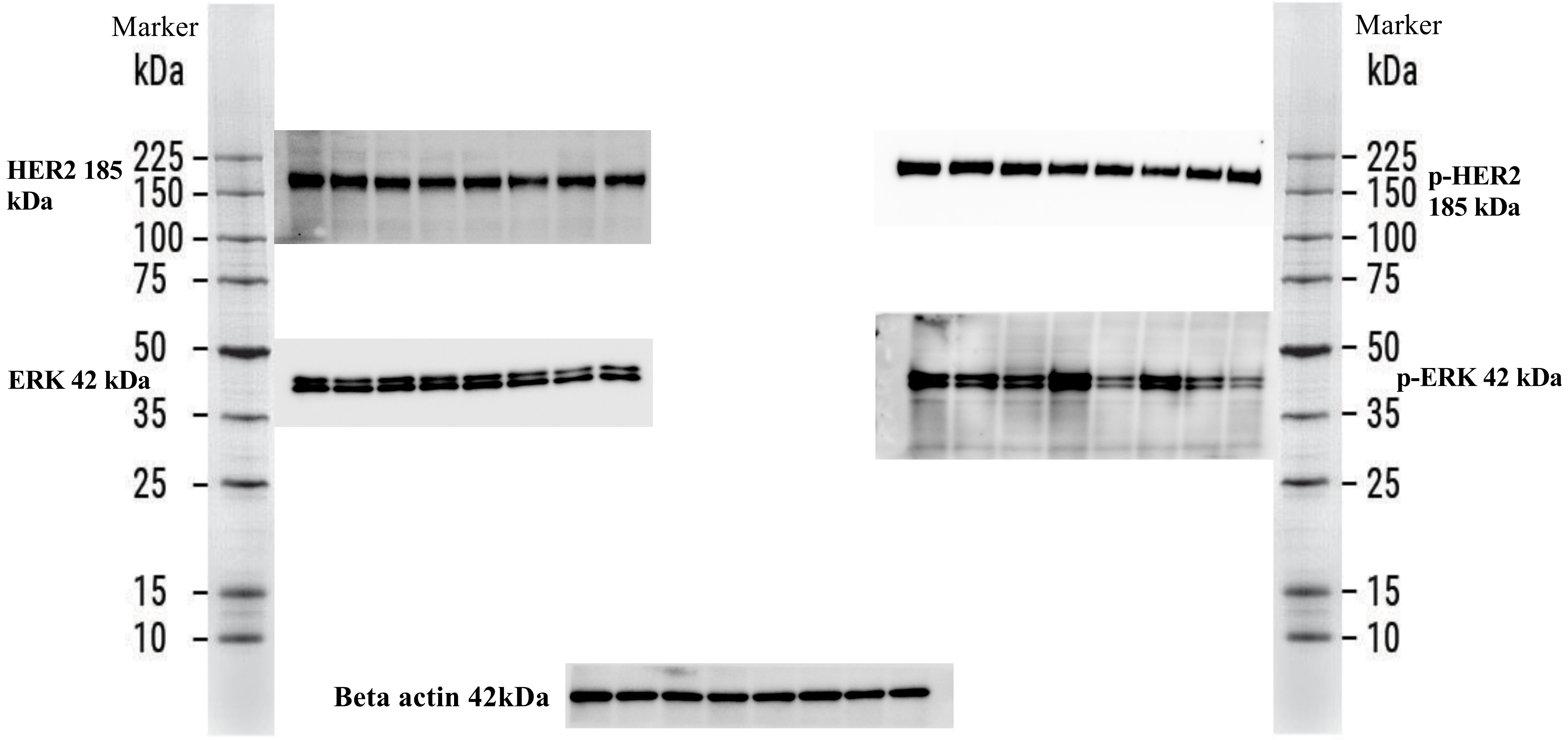

Original western blots from which Figure 8 (Panel A) derived:
analysis of the expression levels of HER2, pHER2, ERK, pERK and β-actin in trastuzumab-resistant BT-474 cells, treated or not with trastuzumab or control-IgG or immune-IgG for 8 hours.

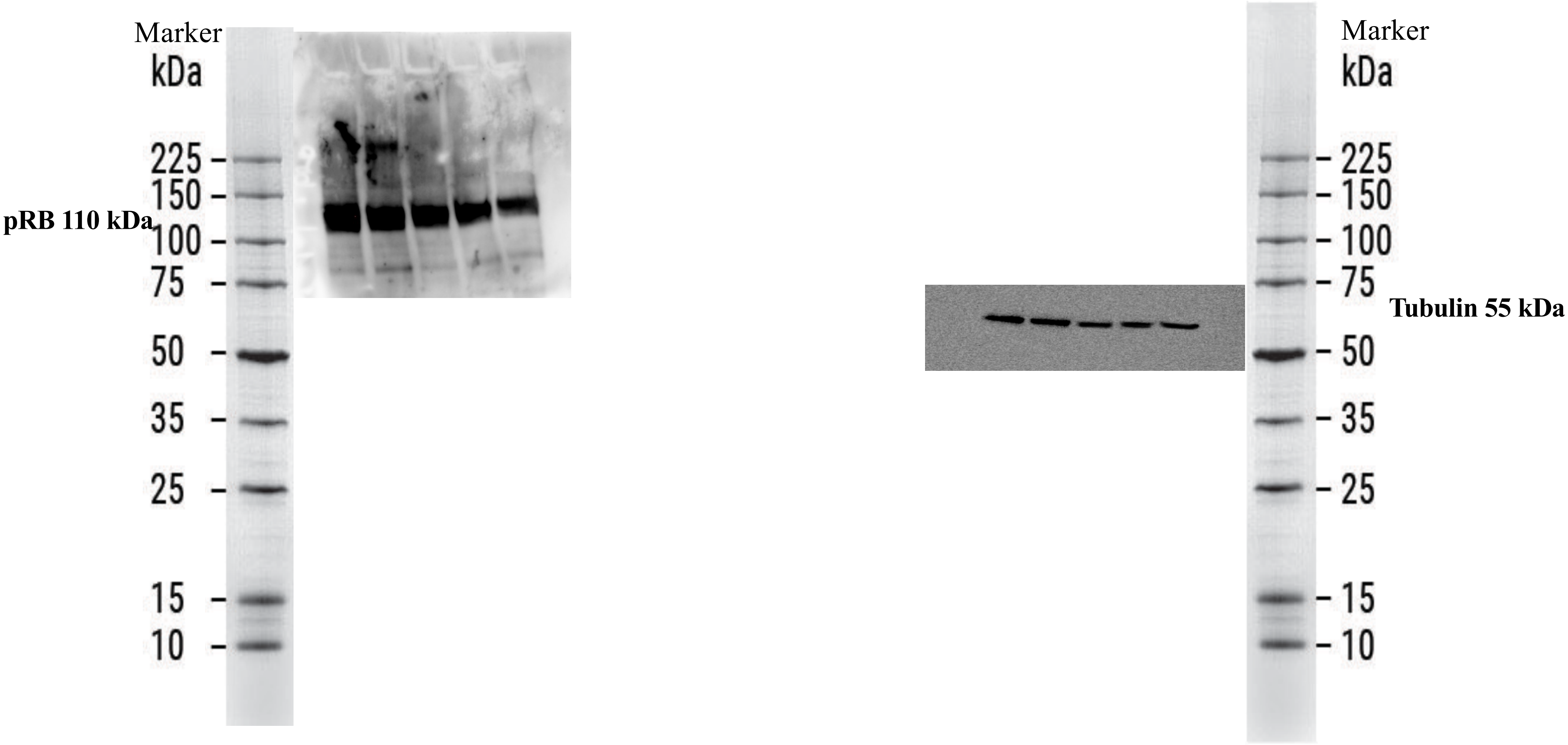

Original western blots from which Figure 8 (Panel C) derived:
analysis of the expression levels of pRB and tubulin in trastuzumab-resistant BT-474 cells, treated or not with trastuzumab or control-IgG or immune-IgG for 8 hours.

	HER2/β-actin	pERK/ERK	pRB/Tubulin
Ctrl	1.679347806	0.992637579	2.357112043
Trastuzumab 15µg/ml	1.362055286	0.842840569	2.467402383
IgG empty15µg/ml	1.285960556	0.92065526	-
IgG empty 30 µg/ml	1.297641049	1.140823538	2.284822489
IgG ECTM 15µg/ml	1.391300748	0.421953155	-
IgG ECTM 30 µg/ml	0.874594002	0.743851765	1.702650828
IgG Δ16ECTM 15µg/ml	1.103172855	0.677053478	-
IgG Δ16ECTM 30µg/ml	1.055927991	0.361036895	1.748787398

Corresponding to Figure 8. Densitometry Readings/intensity Ratio

	BT474				BT474.R				BT474				BT474.R				
Trastuzumab 15µg/ml	-	+	+	+	-	+	+	+	-	+	+	+	-	+	+	+	Trastuzumab 15µg/ml
hours	0	2	8	48	0	2	8	48	0	2	8	48	0	2	8	48	hours
HER2/β-actin	1.9649	1.9889	1.9772	2.1012	2.4800	2.4965	2.5009	2.3058	1.5965	1.4962	1.5098	1.2336	1.7928	1.7502	1.9196	1.7494	p-HER2/β-actin
AKT/β-actin	1.8719	1.6495	2.2231	2.3161	1.4670	1.4061	1.7446	2.3741	2.3843	1.5298	1.7211	1.5484	2.0931	2.1374	1.9948	1.7650	p-AKT/β-actin
Erk/β-actin	1.4184	1.3791	1.5745	1.5142	1.2378	1.2746	1.2001	1.3234	2.1867	1.8896	1.3917	1.0561	1.5239	2.3692	2.1691	2.3872	p-Erk/β-actin

Corresponding to Supplementary Figure S4. Densitometry Readings/intensity Ratio
